# Southern Species From the Biodiversity Hotspot of Central Chile: A Source of Color, Aroma, and Metabolites for Global Agriculture and Food Industry in a Scenario of Climate Change

**DOI:** 10.3389/fpls.2020.01002

**Published:** 2020-07-02

**Authors:** Luis Letelier, Carlos Gaete-Eastman, Patricio Peñailillo, María A. Moya-León, Raúl Herrera

**Affiliations:** ^1^Laboratorio de Fisiología Vegetal y Genética Molecular, Instituto de Ciencias Biológicas, Universidad de Talca, Talca, Chile; ^2^Núcleo Científico Multidisciplinario, Dirección de Investigación, Universidad de Talca, Talca, Chile

**Keywords:** Chilean strawberry, *Fragaria chiloensis* subsp. *chiloensis*, mountain papaya, new crops, *Vasconcellea pubescens*

## Abstract

Two interesting plants within the Chilean flora (wild and crop species) can be found with a history related to modern fruticulture: *Fragaria chiloensis* subsp. *chiloensis* (Rosaceae) and *Vasconcellea pubescens* (Caricaceae). Both species have a wide natural distribution, which goes from the Andes mountains to the sea (East-West), and from the Atacama desert to the South of Chile (North-South). The growing locations are included within the *Chilean Winter Rainfall-Valdivian Forest* hotspot. Global warming is of great concern as it increases the risk of losing wild plant species, but at the same time, gives a chance for usually longer term genetic improvement using naturally adapted material and the source for generating healthy foods. Modern agriculture intensifies the attractiveness of native undomesticated species as a way to provide compounds like antioxidants or tolerant plants for climate change scenario. *F*. *chiloensis* subsp. *chiloensis* as the mother of commercial strawberry (*Fragaria* × *ananassa*) is an interesting genetic source for the improvement of fruit flavor and stress tolerance. On the other hand, *V*. *pubescens* produces fruit with high level of antioxidants and proteolytic enzymes of interest to the food industry. The current review compiles the botanical, physiological and phytochemical description of *F*. *chiloensis* subsp. *chiloensis* and *V*. *pubescens*, highlighting their potential as functional foods and as source of compounds with several applications in the pharmaceutical, biotechnological, and food science. The impact of global warming scenario on the distribution of the species is also discussed.

## Introduction

Few grain species were initially used in human diet. Several other vascular plants species have been incorporated and plant breeding effort have selected inbreeds with interesting and desirable characteristics focus on productivity (Khoshbakh and Hammer, 2008; Şerban et al., 2008). In the case of fruit species, characteristic like, fruit size, color, and texture have been the main quality attributes followed by breeders during the last century, but now a great interest for new traits, such as good aroma, pathogen resistance, high level of antioxidants, healthy compounds, and better postharvest life are considered. Nowadays, fruits from native species could be incorporated in our diet due to their contribution as functional food. In particular, strawberries are rich in secondary metabolites, as well as other attributes, however the fruit is susceptible to several diseases. Hancock et al. (2010) recognizes the importance of octoploid species such as *F*. *chiloensis* and *F*. *virginiana* to enrich traits such as resistance to diseases or better performance against biotic or abiotic stresses in new commercial lines of *F*. × *ananassa*. The global warming scenario of our planet has greatest importance in recent days, as the risk to lose wild plant species is certain due to changes in the ecological conditions (Feng et al., 2010; Lippmann et al., 2019; Moran, 2020). Chile is a world center of origin for cultivated plants and also considered as a biodiversity hotspot. In this sense, two interesting taxa with a history related to modern fruticulture can be mentioned: *Fragaria chiloensis* (L.) Mill. subsp. *chiloensis* (Rosaceae), known as the Chilean strawberry, and *Vasconcellea pubescens* A. DC. (Caricaceae), known as mountain papaya. The present article describes the development, use and potential future for both species.

## Mountain Papaya

**Figure 1:**
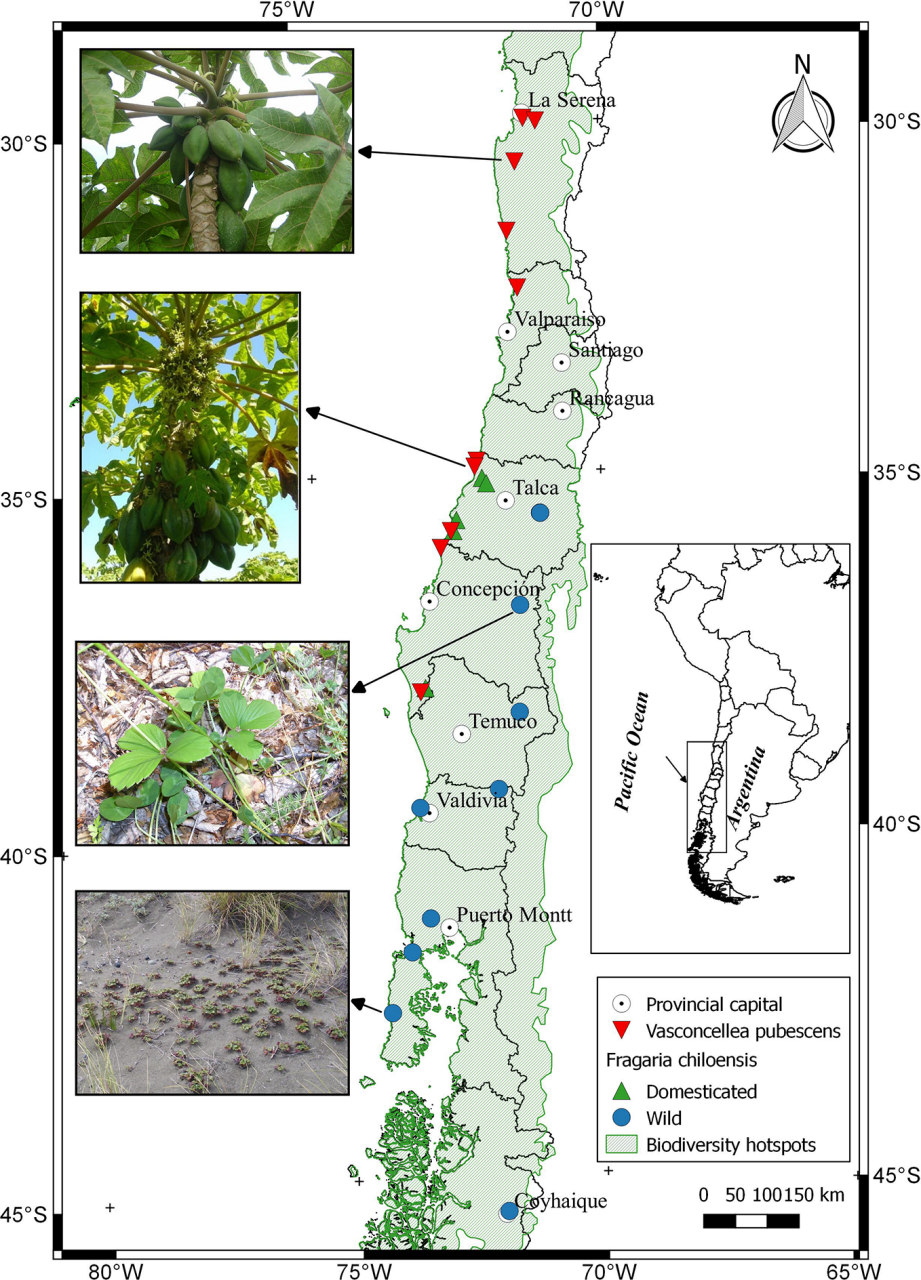
Distribution of *Fragaria chiloensis* (wild and domesticated) and *Vasconcellea pubescens* in Chile. The map shows a partial extension (area) of the *Chilean Winter Rainfall-Valdivian Forests* hotspot. Images located on the left side of the map, from North to South, corresponded to *V. pubescens* plants grown at the localities of Limarí and Lipimávida, and for *F. chiloensis* f. *patagonica* grown at Termas de Chillán and Cucao, respectively.

*Vasconcellea pubescens* A. DC. (Caricaceae) (synonyms: *Carica pubescens* Lenné & K. Koch, *C. candamarcensis* Hook. f., and *V. cundinamarcensis* V.M. Badillo) (Badillo, 2000; Van Droogenbroeck et al., 2002; Hassler, 2019), known as highland or mountain papaya, is a diploid dicotyledonous species (2*n* = 2*x* =18). It is native from subtropical Andean mountains of South America, in particular from low dry mountain forest areas in Colombia and Ecuador (2,000 to 3,000 m altitude). Ecuador, which has 15 of the 21 *Vasconcellea* species, is a center of genetic biodiversity (Van den Eynden et al., 1999; Scheldeman et al., 2011). Some genotypes of *V*. *pubescens* have been well adapted to central Chile before the Spanish colonization, suggesting that its introduction occurred during the Inca Empire or even earlier (Latcham, 1936). A surface of 180 ha of commercial orchards (ODEPA-CIREN, 2019), mostly located between 30° and 33° latitude south (**Figure 1**; **Supplementary Table 1**), highlights Chile as the only country in the southern hemisphere producing the fruit at commercial scale and using it as a source of processed products.

**Figure 2:**
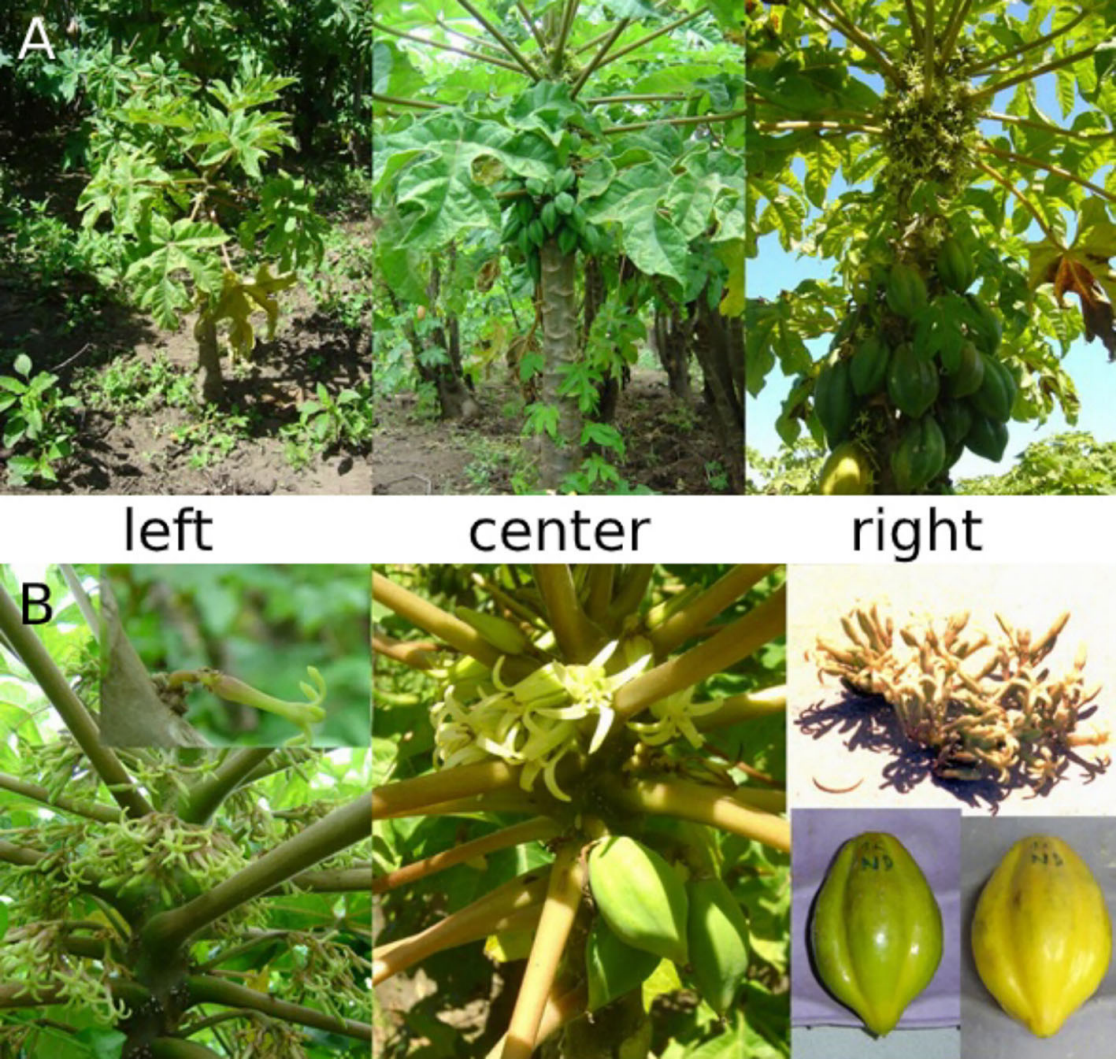
Morphological characteristics of *V. pubescens* tree, flowers and fruit. **(A)** Mountain papaya tree at three growing stages: a two month old tree of half a meter tall producing the first flowers (left); after one year, the tree reaches one meter tall and produces fruit (center); after three years, the tree is two meters tall, it is under full fruit production (right); interestingly, fruit at different developmental stages could be observed in the same tree. **(B)** Sexual forms in *V. pubescens*. Male plants produce staminate flowers, showing their characteristic elongated form, with the stamen inside the flower (left). Female plants produce pistillate flowers, with the characteristic enlargement of the flower base, because of ovary growth (center). Andromonoecious plants produce hermaphrodite and estaminate flowers, where the first ones differentiate from pistillate flowers showing a wider enlargement of flower base (up right). The most notorious change during fruit ripening is the color change of fruit skin, varying from an intense green to bright yellow color (down right).

Mountain papaya is an arborescent plant reaching 1 to 8 m tall, with one central stem and palmate leaves with long petioles at the top (**Figure 2A**). Most of individual plants are dioecious, but a wide range of hermaphrodite phenotype is possible to identify in the field, so formally it is a sub-dioecious species with three sex phenotypes: female, male, and hermaphrodite (**Figure 2B**) (Badillo, 1993). *V. pubescens* presents a case of andromonoecious, where bisexuality is observed in male plants, with a high proportion of male flowers and hermaphrodite ones. Bisexual flowers are male flowers bearing viable ovules leading to the production of small fruits (Scheldeman et al., 2011). Sex in *V. pubescens* is determined by two factors, the Ypm chromosome and *pm* cytoplasmic factor (Horovitz and Jimenez, 1967), which are primitive sex chromosomes and a model for sex evolution determination (Na et al., 2014). The reproductive mode is through seeds, taking between 10 to 12 months to reach reproductive age, and maintained for 5 years at commercial scale. Then, plants progressively produce less fruit and with lower quality, nevertheless, plants older than 20 years are found in Chilean orchards. Mountain papaya tree shows a slow growth, with a continuous flower and leaf production; when lower leaves get older they fall into the ground (Sánchez, 1994).

*V. pubescens* is sensitive to cold, stem and leaves could be affected leading to complete plant death when temperatures fall below 2°C; even ripening of the fruit could be altered by cold. Cold episodes have been more frequent in Chile in recent years, even in coastal areas where chilling injuries on commercial orchards were absolutely absent 10 years ago. Additionally, extended drought stress promotes the continuous loss of leaves (Sánchez, 1994).

**Figure 3:**
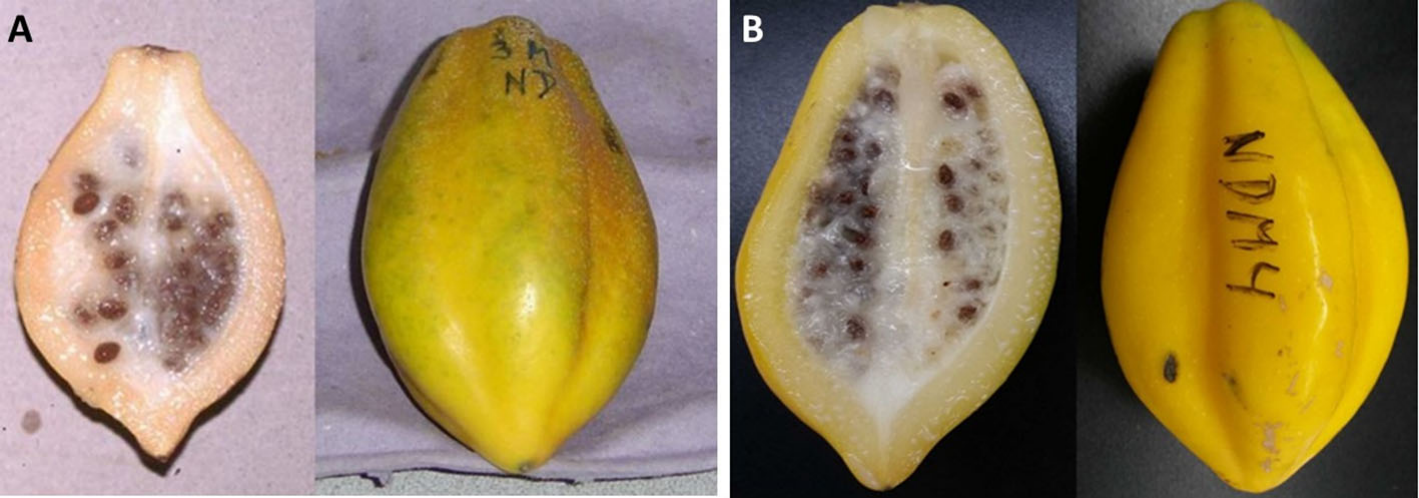
Morphological characteristics of mountain papaya fruit at the ripe stage. Fruit shows its characteristic shape, thin pulp layer, with an inside cavity full of maternal tissue that contains many seeds (left), and the typical yellow skin color of ripe fruit with five ridges (right). The fruit was harvested at Lipimávida in seasons 2010 **(A)** and 2018 **(B)**.

Mountain papaya tree bears fruit at the leaf axis spirally arranged along the trunk, so it is possible to observe from immature (small size, green colour) to ripe (big size, bright yellow) fruit stages in one single plant. In natural populations, birds eat ripe and overripe fruits, allowing seed dispersal and germination. Seeds have a high germination rate (60% in 30 days) without the requirement of a dormancy period (Sánchez, 1994). The fruit is oblong, truncated at the base, with five pronounced ridges; a ripe fruit is about 8–15 cm long, 5–6 cm diameter, and 200 g mean weight (García, 1975) (**Figure 3**). The fruit has a juicy yellow flesh with a strong and aromatic flavor, characterized by a high content of vitamins (A, B, and C), high level of antioxidants, and a milky latex (highly abundant in immature stages) that contains a mixture of proteolytic enzymes, commonly named as papain (Van Droogenbroeck et al., 2002; Moya-León et al., 2004). The fruit is commonly used to prepare preserves, jam, sweet candies and nectar, meanwhile its latex is widely used in the industry as meat tenderizer. Mountain papaya is frequently compared with the common and worldwide known papaya (*Carica papaya* L.), being *V. pubescens* smaller and less succulent than *C*. *papaya* but with a greater aroma and flavor (Van den Eynden et al., 1999; Scheldeman et al., 2003; Van den Eynden et al., 2003).

### Diversity and Genetic Structure

Some phenotypic variations are observed in mountain papaya, mainly in plant height, number of branches and fruit size. Nevertheless, the most important differences are in sex determination. Although no environmental variation can be observed for pistillate and staminate plants, the proportion of male and hermaphrodite flowers in andromonoecious plants depends on climate conditions. On the other hand, the incompatibility barrier is labile in *Vasconcellea*, and therefore, this allows the possibility to increase genetic variation through the formation of spontaneous hybrid specimens between different species (Badillo, 1993; Sánchez, 1994). Interspecific hybrids have been found in natural populations (De Zerpa, 1959), however only one has commercial success: the babaco (*Vasconcellea* × *heilbornii* (V.M. Badillo) V.M. Badillo). Babaco results from the cross between *V. pubescens* and *V. stipulata* (V.M. Badillo) V.M. Badillo (Horovitz and Jimenez, 1967; Badillo, 1971; Scheldeman et al., 2011). Molecular studies using molecular markers suggest that the origin of *V*. × *heilbornii* results of a complex evolution process where *V. pubescens*, *V. stipulata*, and *V. weberbaueri* (Harms) V.M. Badillo are parental species (Scheldeman et al., 2011). Molecular analysis also confirms hybrids obtained by the cross of *V. pubescens* and *V. monoica* (Desf.) A. DC. (Kyndt et al., 2005; Kyndt et al., 2006) and interspecific crosses with *V. stipulata*, *V. monoica*, *V. microcarpa* (Jacq.) A. DC. and *V. horovitziana* (V.M. Badillo) V.M. Badillo (Horovitz and Jimenez, 1967; Badillo, 1971).

A high genetic diversity along the natural distribution pattern in the centres of origin (Ecuador and northern Perú) is assumed, but scarce information exists regarding the history and genetic diversity of *V. pubescens* introduced in Chile. A low genetic variability but a high genetic polymorphism was reported using ISSR markers (Carrasco et al., 2009). Based on the results, the authors suggested that few introduction events of genetic material from the original populations were concreted during time and this material constitutes the basal genotype of Chilean material.

### Phytochemistry

Aroma is one of the most important attributes of mountain papaya fruit and it is due to a complex mixture of volatile compounds produced by the fruit (Balbontín et al., 2007). Main volatile compounds produced by the fruit are esters and alcohols (linear and branched), and their production increase as ripening progresses (Balbontín et al., 2007). Most of esters identified in mountain papaya fruit are potent odour compounds, such as ethyl butanoate, ethyl acetate, ethyl hexanoate, and ethyl 2-methylbutanoate, and the most important alcohol is butanol. The dynamic of their production during ripening depends on ethylene, which agrees to consider mountain papaya as a climacteric fruit (Moya-León et al., 2004; Balbontín et al., 2007). Several other volatile compounds such as methyl cis-hex-3-enoate, isopentyl acetate, methyl 3-hydroxyhexanoate and ethyl nicotinoate have been reported in mountain papaya fruit grown in Chile, however they have not been detected in fruit grown in Colombia (Morales and Duque, 1987). On the other hand, many fruits accumulate antioxidant activity during ripening, which is a desirable feature for functional foods. Active antioxidant phenolics from mountain papaya fruit include quercetin glycosides, rutin, and manghaslin, which are not produced by common papaya (*C. papaya*) (Simirgiotis et al., 2009). This suggests a metabolic divergence between both plant species.

All members of the Caricaceae family have laticifers and produce milky latex when a plant tissue is damaged. This latex helps as a defensive mechanism against predators and to heal wounded sites (Konno et al., 2004). The latex composition and proteinase quantity is different between species. The latex from *C. papaya* has been described and it consists of a mixture of hydrolase enzymes like quitinases and proteases, being the most characteristics papain, chymopapain, caricain (proteinase Ω) and glycil endopeptidase (proteinase IV) (El Moussaoui et al., 2001). There are some evidences showing that freeze-dried latex from *V. pubescens* has 5 to 8 times more proteolytic activity compare to the one obtained from *C. papaya* (Baeza et al., 1990). On the other hand, the extraction of *V. pubescens* latex and its separation into four different fractions named as CC-I to CC-IV was reported (Walraevens et al., 1993); two of them, CC-I and CC-III have been characterized and corresponded to papain and chymopapain, respectively. The mixture of latex proteinases has been used to treat gastric ulcers in rodent models (Mello et al., 2008). More recently, two different fractions were obtained from *V. pubescens* latex: a fraction showing high proteinase activity (CMS) and a fraction showing moderate to low proteinase activity (Teixeira et al., 2008). On the other hand, the fraction called P1G10 has been used for gastric ulcers and diabetic foot treatments in several wounded models (Tonaco et al., 2018). At the same time, the reduction of tumour mass in animals bearing melanoma and metastasis level was observed using this fraction (Lemos et al., 2018).

## The Chilean Strawberry

*Fragaria* L. is a member of the Rosaceae family. There are about 20 species distributed in temperate Eurasia and North and South America. Staudt recognizes four subspecies for *F*. *chiloensis* (L.) Mill. based on morphological traits with a worldwide distribution: two of them are located in North America (*F*. *chiloensis* subsp. lucida (E.Vilm. ex J. Gay) Staudt, and *F*. *chiloensis* subsp. *pacifica* Staudt), one in Hawaii (*F*. *chiloensis* subsp. *sandwicensis* (Decne.) Staudt) and the last one in South-America (i.e., Ecuador, Bolivia, Perú, Argentina and Chile) (*F*. *chiloensis* subsp. *chiloensis* Staudt*)* (Staudt, 1962; Bringhurst, 1990; Staudt, 1999). Staudt also proposed two botanical forms for the subspecies *F*. *chiloensis* subsp. *chiloensis* based on characters such as plant size, texture of leaves, color and fruit size: *F. chiloensis* (L.) Mill. subsp. *chiloensis* f. *chiloensis* Staudt, is a landrace that produces large fruit with white/pink receptacle and white flesh; and *F. chiloensis* (L.) Mill. subsp. *chiloensis* f. *patagonica* Staudt, that produces small red fruits (Staudt, 1962; Staudt, 1999). Shortly after the conquest of the Central-South part of Chile, location where the Mapuches used to live, *F. chiloensis* subsp. *chiloensis* was taken as a war trophy by the Spaniards and the species was introduced in Perú. Historical reports indicate that Garcilaso de la Vega in 1557 observed a new strawberry species in the land and markets of Cuzco City, different in size compared to the European species (Popenoe, 1921). Thereafter, the species was introduced in Ecuador by the Spaniards; although there are not exact records, it seems to be before 1789 (Popenoe, 1921). Interestingly, it was not until 1712 that *F. chiloensis* subsp. *chiloensis* was introduced in Europe by some explorers coming from Chile (Popenoe, 1921; Darrow, 1966; Liston et al., 2014). From an agronomic perspective, mainly in Chile and in minor scale in Perú and Ecuador, *F. chiloensis* subsp. *chiloensis* was cultivated as a fruit source during the first half of 1900 century, however when new cultivars reached from Europe and USA, the species was displaced and its cultivation was not favoured (Finn et al., 2013).

**Figure 4:**
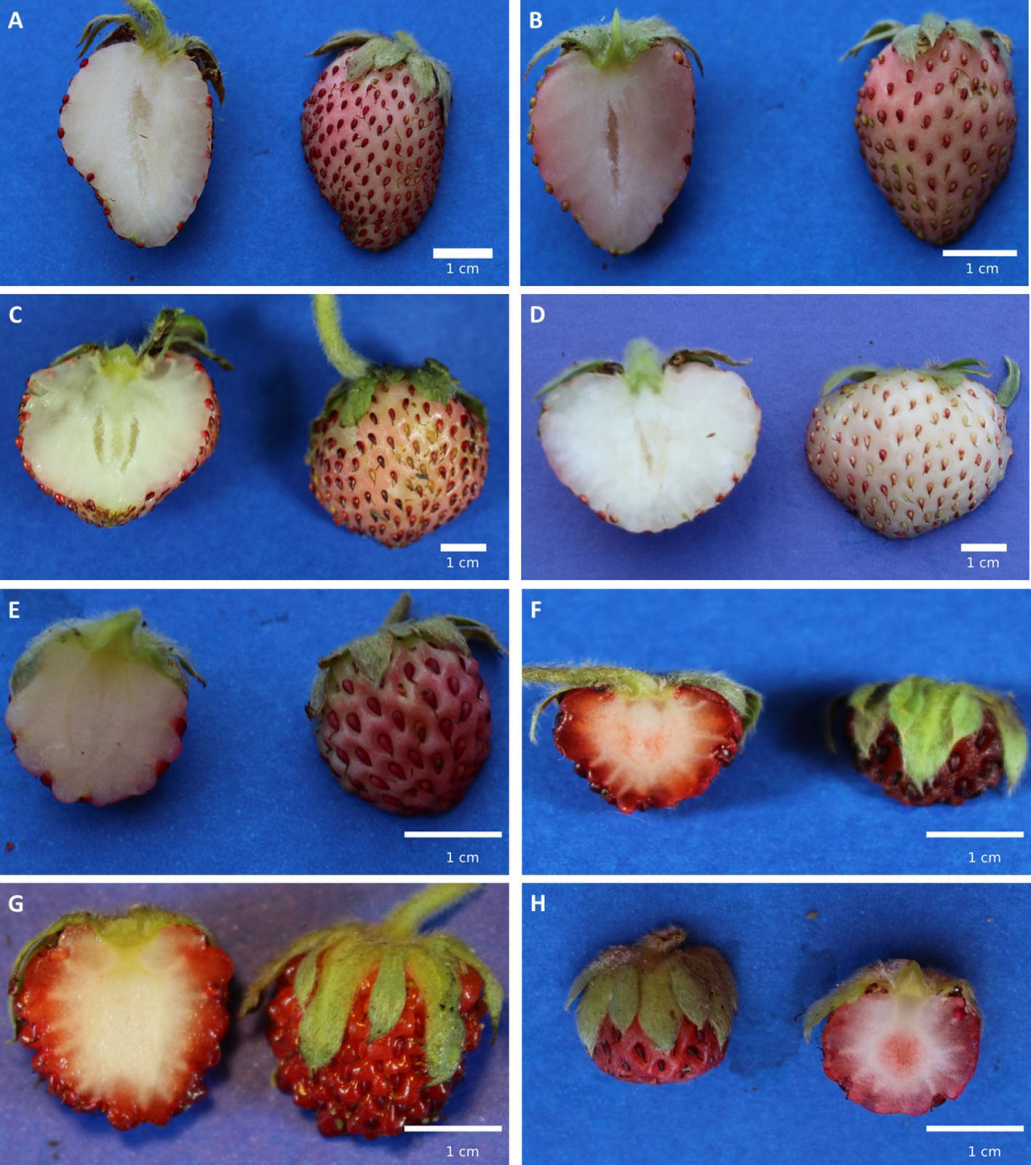
Morphological characteristics of Chilean strawberry fruit at the ripe stage. **(A-D)** correspond to *Fragaria chiloensis* f. *chiloensis* fruit distributed from North to South, from the localities of Huelón Alto, Pelluhue, Purén and Contulmo, respectively. **(E-H)** correspond to wild *F. chiloensis* fruit distributed from North to South, from the localities of Termas de Chillán (see **Figure 1**), Curiñanco, Cuesta Gutiérrez and Cucao (see **Figure 1**), respectively.

In botanical terms, *F. chiloensis* subsp. *chiloensis* is a perennial herb, with strong and well developed runners; trifoliate leaves; with unique or few flowers, which can be dioic or hermaphrodite. The berry is an aggregate fruit, which develops from a flower that contains several ovaries (Marticorena, 2019). The fertilized ovaries develop into achenes that resulted embedded on surface of the receptacle. The edible receptacle or thalamus is usually considered as the most attractive part in sensory terms (**Figure 4**).

Nowadays in Chile, the two botanical forms of *F. chiloensis* subsp. *chiloensis* grow in central-south part of Chile (**Figure 1**), and can be found between the O´Higgins and the Aysén Regions (35°05'– 45°32' latitude South) (Lavin et al., 2000; Rodríguez et al., 2018), growing from forests (understory) to open vegetation environments (Kalkman, 2004) (**Figure 1**). These areas present contrasting conditions for plant development, with differences in light, temperature and nutrients availability (Moreira-Muñoz, 2011). The fruit phenotype of Chilean accessions is quite diverse. As a way to demonstrate this, plants collected from different geographic areas were established in one edaphoclimatic condition (Linares) (**Supplementary Table 1**). Cultivated *F. chiloensis* f. *chiloensis* species from Huelón Alto, Pelluhue, Purén, and Contulmo produces fruit of white flesh, white/pink receptacle of uniform medium size (**Figures 4A–D**). On the contrary, the fruit produced by wild plants of *F. chiloensis* f. *patagonica* collected in Termas de Chillán, Curiñanco, Cuesta Gutiérrez and Cucao produce small fruit size with red receptacle (**Figures 4E–H**). The white flesh fruit is commercially produced at small scale and its main use is for fresh consuming or to prepare beverages (Pardo and Pizarro, 2013).

*F*. *chiloensis* f. *chiloensis* (white fruit) is considered as one of the parentals for the commercial strawberry (*F*. × *ananassa* (Duchesne ex Weston) Duchesne ex Rozier) along with *F*. *virginiana* Mill., which have also been supported by molecular analysis (Njuguna et al., 2013; Tennessen et al., 2014; Edger et al., 2019). *F*. *chiloensis* f. *chiloensis* has been widely studied particularly for its distinctive exotic white-pink color and characteristic aroma (Hancock et al., 1999; Retamales et al., 2005; González et al., 2009b). Several studies have been published reporting the softening of this fruit, showing that when firmness is reduced cell wall polymers are broken down (Figueroa et al., 2008; Moya-León et al., 2019). Several genes and enzymes involved in cell wall disassembly have been characterized (Figueroa et al., 2008; Figueroa et al., 2009; Pimentel et al., 2010; Opazo et al., 2013; Méndez-Yáñez et al., 2017; Méndez-Yáñez et al., 2020). Softening requires the fine coordination of several molecular activities, including the participation of plant hormones and transcription factors (Handford et al., 2014; Carrasco-Orellana et al., 2018; Moya-León et al., 2019). The key role played by the molecular coordinators on the expression of cell wall degrading genes impacts softening and the postharvest quality of the fruit.

The plant and fruit of *F*. *chiloensis* f. *chiloensis* show tolerance to pathogens and diseases (González et al., 2013). In addition, the plant species has the ability to grow under different abiotic stress conditions (low temperatures, salty soils) which gives a great potential for breeding purposes (González et al., 2009a; Aceituno-Valenzuela et al., 2018). In this sense, the species can be considered as genetic source for strawberry breeding programs.

*F*. *chiloensis* subsp. *chiloensis* is located in one of the worldwide biodiversity hotspots. A hotspot is defined as an area with high diversity of endemic species (over 1,500), where 30% or less of this area should be threatened, and there are 34 areas recognized globally (Arroyo et al., 1999; Myers et al., 2000; Mittermeier et al., 2004). The *Chilean Winter Rainfall-Valdivian Forests* hotspot covers a great part of northern and southern Chile and contains 1,957 different endemic species (Mittermeier et al., 2004). A reduction of plant communities have been observed directly by a high change in soil use from forests to forest plantations or agriculture (Arroyo et al., 1999). Several species belong to this area, which are used for human consumption (**Table 1**). Climate change models predict for the geographic area of this hotspot a decrease in the distribution range of plant species, in addition to, distribution displacements towards the south or to take refuge in the Andes Mountains (Pliscoff et al., 2012; Bambach et al., 2013).

**Table 1:** Native plants from the Chilean winter rainfall-Valdivian forest hotspot with potential in agriculture.

Native plants	Edible organ	Folk name	Family
*Araucaria araucana* (Molina) K. Koch	Fruit	Pehuen	Araucariaceae
*Aristotelia chilensis* (Molina) Stuntz	Fruit	Maqui	Elaeocarpaceae
*Berberis microphylla* G. Forst.	Fruit	Calafate	Berberidaceae
*Eulychnia acida* Phil.	Fruit	Copao	Cactaceae
*Gaultheria* species	Fruit	Chauras	Ericaceae
*Geoffrea decorticans* (Gillies ex Hook. et Arn.) Burkart	Fruit	Chañar	Fabaceae
*Gevuina avellana* Molina	Fruit	Avellano	Proteaceae
*Gomortega keule* (Molina) Baill.	Fruit	Queule	Gomortegaceae
*Gunnera tinctoria* (Molina) Mirb.	Petiole	Pangue	Gunneraceae
*Lardizabala biternata* Ruiz et Pav.	Fruit	Cóguil	Lardizabalaceae
*Peumus boldus* Molina	Fruit	Boldo	Monimiaceae
*Prosopis chilensis* (Molina) Stuntz emend. Burkart	Fruit	Algarrobo	Fabaceae
*Prumnopitys andina* (Poepp. ex Endl.) de Laub.	Fruit	Lleuque	Podocarpaceae
*Puya chilensis* Molina	Vegetative buds	Puya	Bromeliaceae
*Ribes magellanicum* Poir.	Fruit	Zarzaparrilla	Grossulariaceae
*Rubus geoides* Sm.	Fruit	Miñe miñe	Rosaceae
*Solanum tuberosum* L. subsp. *tuberosum*	Tuber	Papa chilota	Solanaceae
*Ugni molinae* Turcz.	Fruit	Murtilla	Myrtaceae

### Diversity and Genetic Structure

A center of diversity assures sustainable genetic improvement for plant species. The wide range of environmental conditions covered by the Chilean hotspot can be used as a source of genetic adaptation to different habitats, conferring tolerance to several stresses or environmental factors. Besides, the loss of genetic diversity within a species can result in the loss of useful and desirable traits and may eliminate options to use untapped resources for food production, industry and medicine (Hoisington et al., 1999; Govindaraj et al., 2015).

The genetic diversity in *Fragaria* has been studied using molecular markers such as microsatellites (SSR) or dominant markers (ISSR, AFLP and RAPD), and more recently using a high throughput system such as single nucleotide polymorphisms (SNPs) array (Bassil et al., 2015; Hilmarsson et al., 2017; Jung et al., 2017). SSR markers, for example, were used with the purpose to identify *F*. × *ananassa* cultivars (Monfort et al., 2006; Shimomura and Hirashima, 2006; Honjo et al., 2011). The IStraw90 Axiom array is a genotyping platform for *Fragaria* able to identify SNPs, indels, and also is used for mapping (Mahoney et al., 2016; Nagano et al., 2017; Sooriyapathirana et al., 2019; Oh et al., 2020). In the case of *F. chiloensis* subsp. *chiloensis* a small number of accessions were analyzed and low genetic diversity and no genetic structure was reported (Carrasco et al., 2007; Oñate et al., 2018). The vegetative propagation of the species was used as argument for the low genetic variability found along Chile (Lavin et al., 2000), however despite of that, a breeding and improvement program on the species was considered feasible. Several crosses of the Chilean strawberry and *Fragaria* spp have been performed which have been evaluated for the generation of elite genotypes (Hancock et al., 2001; Finn et al., 2013). Recently, new hybrids were successfully obtained by crossing four different *F*. × *ananassa* cultivars with *F. chiloensis* f *chiloensis*, increasing diversity and facilitating new bridge species for breeding (Luque et al., 2019). The selection from the breeding programe can be facilitated and optimized by the use of molecular techniques (Edger et al., 2019; Oh et al., 2019).

### Phytochemistry

*F*. *chiloensis* subsp. *chiloensis* fruit contains an interesting composition of phenolics and high antioxidant activity. Ellagic acid and cinnamic acid glycosides are present in the white Chilean strawberry fruit (Cheel et al., 2005), and the fruit is also rich in phenolic antioxidants (Cheel et al., 2007; Simirgiotis et al., 2009). In addition, the fruit is characterized for having great aroma properties (González et al., 2009b). Differences in the volatile profiles have been described among commercial strawberry cultivars (Staudt et al., 1975; Forney et al., 2000), and most importantly between cultivated and wild species, being wild species those with the highest concentration of volatiles and better aroma properties (Ulrich et al., 2007). Commercial strawberries produce numerous volatile compounds including esters, aldehydes, ketones, alcohols, terpenes, furanones, and sulfur compounds (Latrasse, 1991; Zabetakis and Holden, 1997), nevertheless esters are the most abundant class of compounds (25% to 90% of total volatiles) (Pérez et al., 1992), and provide the fruity notes of fresh ripe fruit (Pyysalo et al., 1979; Schreier, 1980). Some esters identified in the white Chilean strawberry fruit include ethyl acetate, methyl butanoate, 2-methyl acetate, octyl acetate, octyl butanoate, hexyl acetate, ethyl heptanoate, 2-hexenyl butanoate, benzyl acetate, and hexyl 2-methyl butanoate, which were also described in several *F*. × *ananassa* cultivars (Pyysalo et al., 1979; Zabetakis and Holden, 1997; Azodanlou et al., 2003; Berna et al., 2007; González et al., 2009b). Importantly, esters such as hexyl propanoate, ethyl 4-decenoate, 2-phenylethyl propanoate, and ethyl 2,4 decadienoate have been identified in *F. chiloensis* f. *chiloensis* and not in *F*. × *ananassa* (González et al., 2009b).

Anthocyanins are the color pigments of strawberry fruit. In red strawberries glycosylated anthocyanins derived from pelargonidin and cyanidin are normally present, being pelargonidin 3-glucoside the most abundant anthocyanin (Tulipani et al., 2008). In the white Chilean strawberry, the main anthocyanin is cyanidin 3-O-glucoside, which is mainly present in the achenes (Cheel et al., 2005; Salvatierra et al., 2010; Salvatierra et al., 2014). Importantly, anthocyanins have potential benefits and broad health-promoting effects for human considering the antioxidant activity (Chamorro et al., 2019; Hwang et al., 2019; Mazzoni et al., 2019).

The potential health benefits of strawberry fruit consumption have been analyzed, and some *in vivo* effects on the oxidative status in human and animal models are now available. Chilean white fruit aqueous extracts have a protective effect on platelet aggregation (Parra-Palma et al., 2018) and can exert protection on human epithelial gastric cells against free radical-induced damage (Avila et al., 2017). On the other hand, dietary supplementation of rats with white Chilean strawberry juice favors the normalization of oxidative and inflammatory effects in response to a liver injury induced by lipopolysaccharides (Molinett et al., 2015). These results support the idea to considerate the Chilean strawberry fruit as a functional food.

## Mediterranean Crop and Climate Change

**Figure 5:**
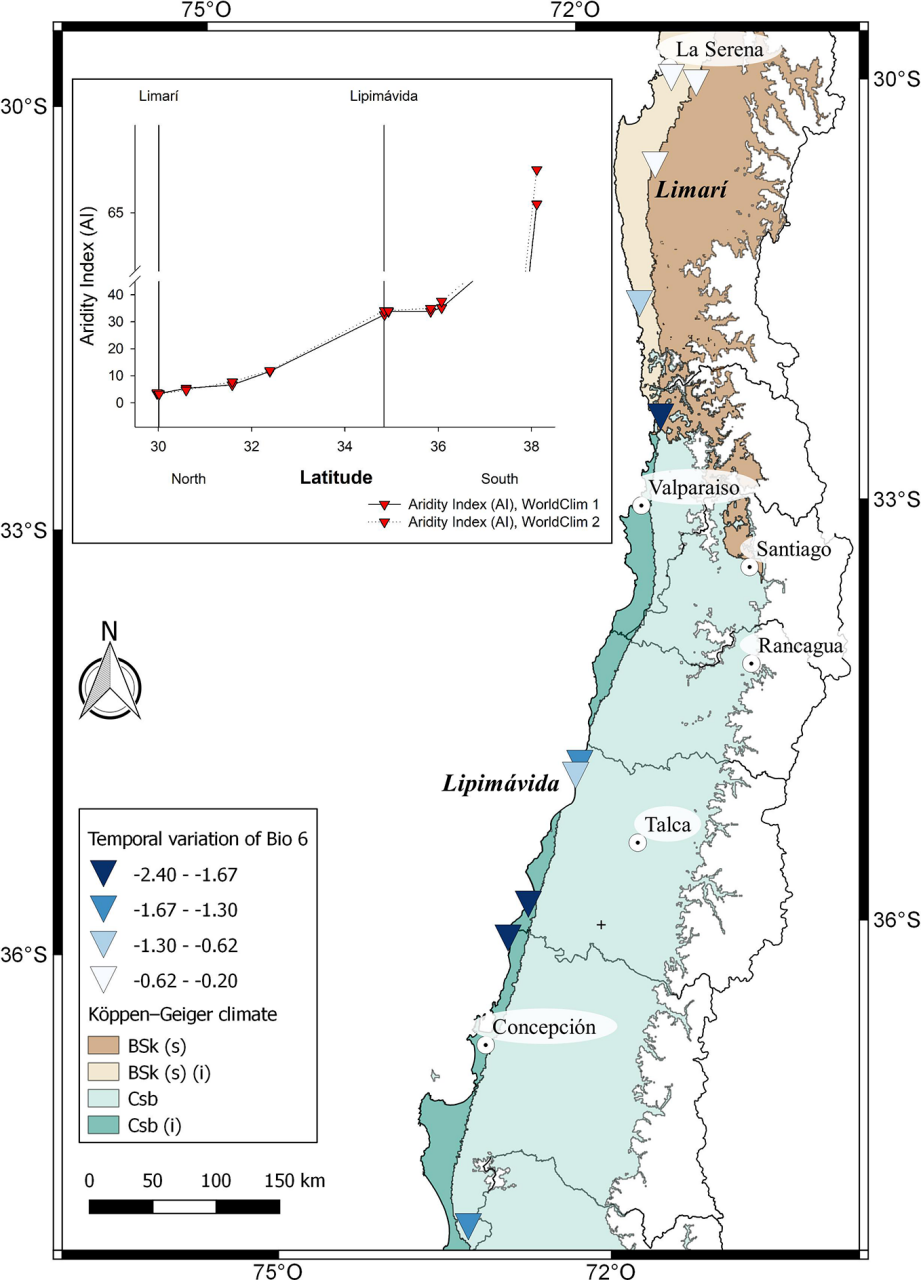
Climatic characterization of cultivated localities of *V. pubescens* distributed from North to South. The comparison of Bioclimatic variable 6 (Min temperature of coldest month) between WorldClim 1 and 2 for each locality is shown in the map. In the insert, De Martonne Aridity Index (AI) for each locality. On the X axis, the localities of Limarí and Lipimávida are marked as reference, as previously shown in **Figure 1**.

**Figure 6:**
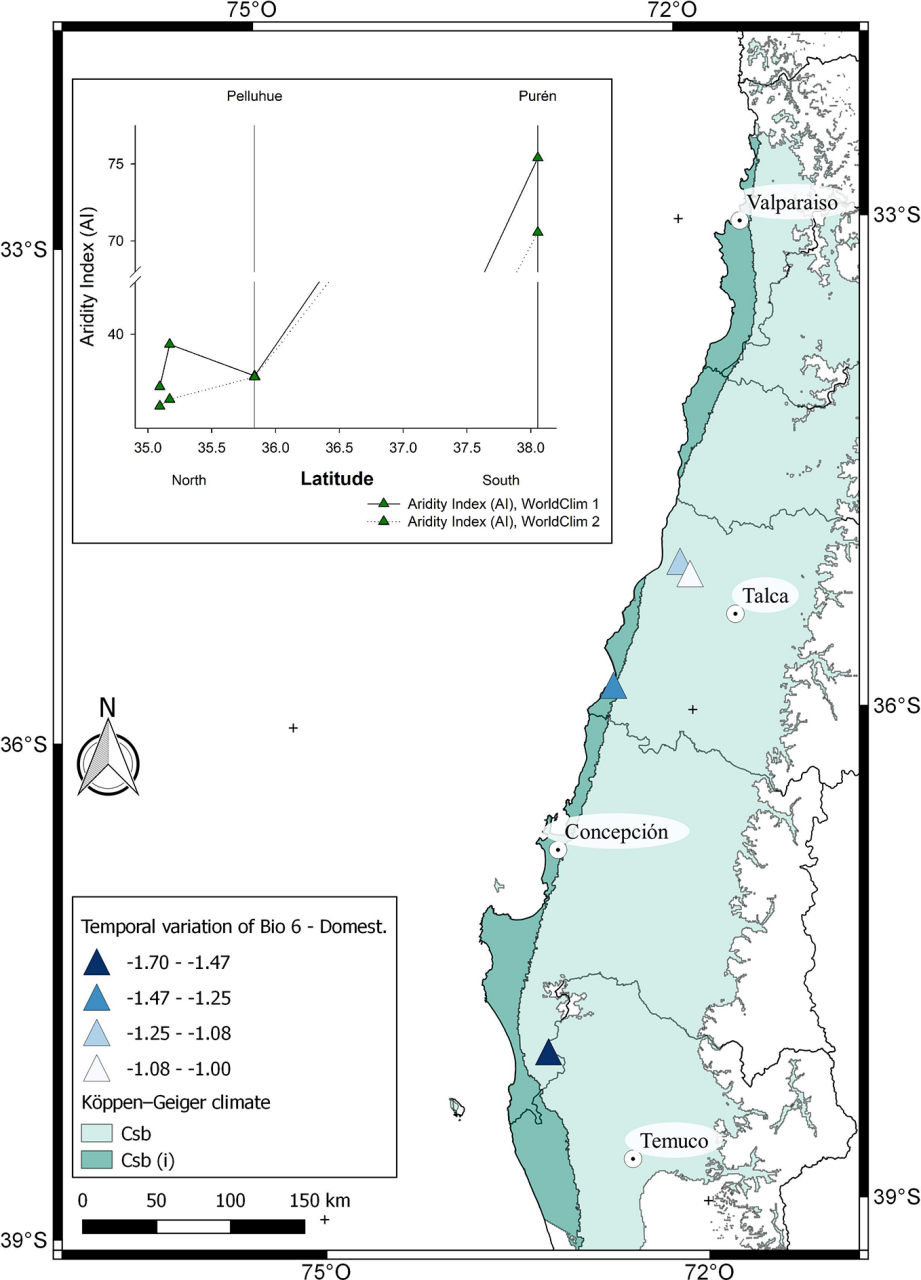
Climatic characterization of the localities where *F. chiloensis* f. *chiloensis* is cultivated in Chile and distributed from North to South. The comparison of Bioclimatic variable 6 (Min temperature of coldest month) between WorldClim 1 and 2 for each location is shown in the map. In the insert, De Martonne Aridity Index (AI) for each locality.

**Figure 7:**
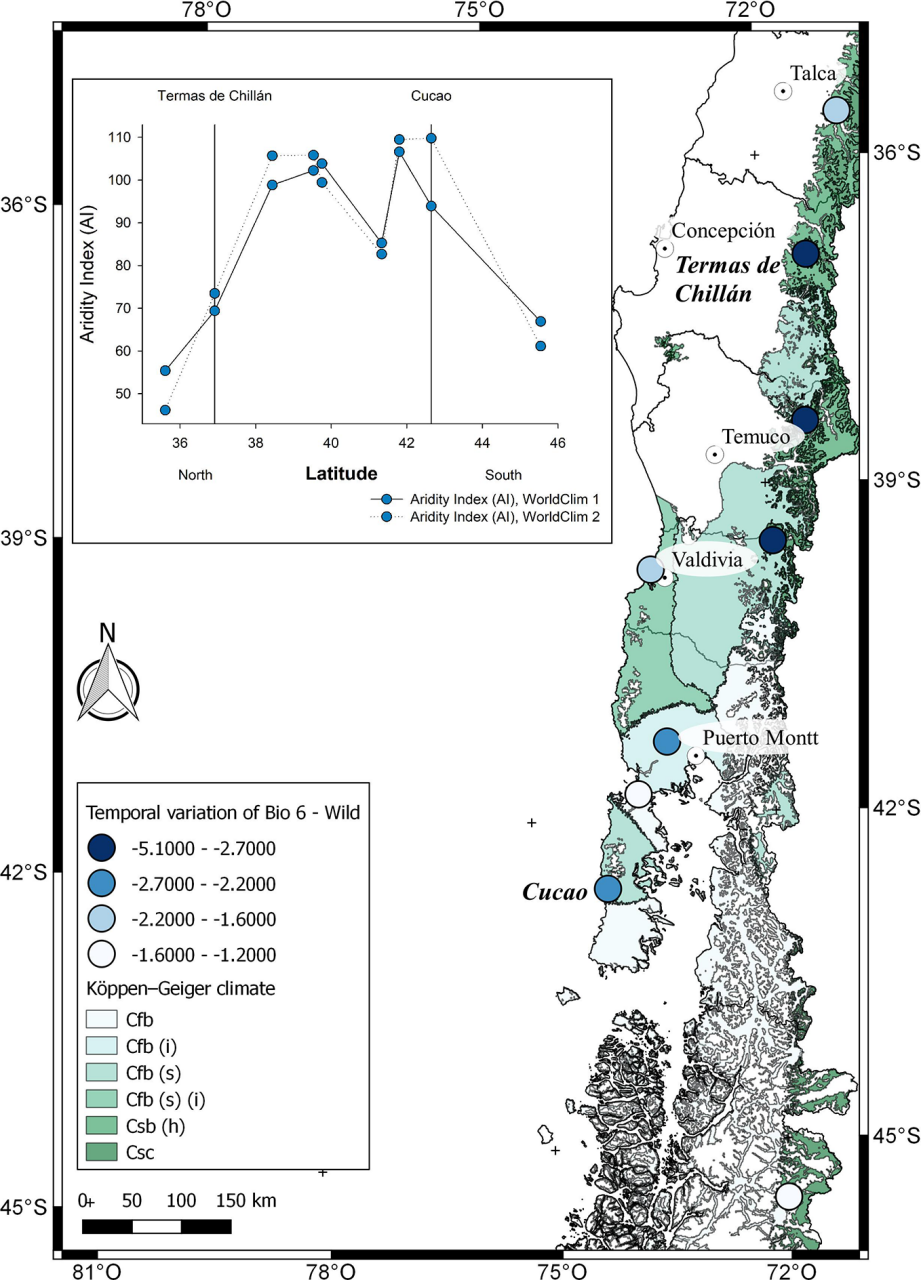
Climatic characterization of the localities where *F. chiloensis* f. *patagonica* is distributed in Chile, from North to South. The comparison of Bioclimatic variable 6 (Min temperature of coldest month) between WorldClim 1 and 2 for each locality in which *F. chiloensis* f. *patagonica* grows are shown in the map. In the insert, De Martonne Aridity Index (AI) for each locality. On the X axis, the localities of Termas de Chillán and Cucao are marked as reference, as previously shown in **Figure 1**.

In general, papaya plants grow mostly in tropical and subtropical Andes. In Chile, as shown in **Figure 1**, mountain papaya (*V*. *pubescens*) is cultivated in coastal areas between latitudes 30° to 36° South, which corresponds to a climate with subtropical influence characterized by moderate thermal ranges and low occurrences of frosts. Recently, Sarricolea et al. (2017) carried out a climatic classification of Chile, and proposed that the cultivated populations of *V*. *pubescens* could be cultivated in 4 climatic types of Köppen-Geiger (see **Figure 5** and **Supplementary Table 1**). On the other hand, regarding the cultivation of strawberry in Chile, both *F*. × *ananassa* and *F. chiloensis* f. *chiloensis* grow between latitudes 32° to 37° South, in coastal areas of Chile, where climate corresponds to a Mediterranean type, with 8 variants according to Köppen-Geiger climate classification (see **Figures 6** and **7** and **Supplementary Table 1**, Sarricolea et al., 2017).

The Intergovernmental Panel on Climate Change (IPCC) predicts that mid-latitude areas would be affected by the increment in drought, with irregular rainfall regimes and rising temperatures (Parajuli et al., 2019). These locations are the cultivation areas where main crops are worldwide concentrated, which also include our both species under study. The average temperature and precipitation within the area of cultivation for *V*. *pubescens* and *F*. *chiloensis* can be estimated using the information provided by climate models such as WorldClim. WorldClim 1 considers the interpolation of observed climatic data representative for the period of 1960–1990 (Hijmans et al., 2005), and WorldClim 2 considers climatic data between 1970-2000 (Fick and Hijmans, 2017). Although WorldClim defines 19 bioclimatic variables, only Annual Mean Temperature (BIO 1), Min Temperature of Coldest Month (BIO 6) and Annual Precipitation (BIO 12) were used in this analysis as they provide robust differences between sites. Aridity index (AI) was determined using BIO1 and BIO12, which correlates the precipitation (annual or monthly) with mean temperature (also annual or monthly), and allows the classification of study sites as desert, meadow or forest (Wang and Takahashi, 1999). High aridity index values indicate a site with more water availability. Considering that the increase in frosts could affect crops in mid-latitude areas BIO 6 variable was also considered (Parajuli et al., 2019).

In the case of *V. pubescens*´s distribution, the model predict a similar precipitation behavior for each location along the years, with values from North to South ranging from 90 mm average in Limarí to 1,515 mm average at the southern end of the distribution (Lleu-Lleu) (**Supplementary Figure 1**). Interestingly, Squeo et al. (1999) indicated that the annual rainfall for the area of La Serena (north of Limarí) was reduced from 180 mm at the beginning of 1900 to about 80 mm by the year 2000, which evidences a precipitation drop of 40% during the last century. On the other hand, a slight decrease in the average annual temperature (BIO1) and a drop in the minimum temperature for the coldest month by at least 2.4 °C can be observed (**Figure 5**, **Supplementary Figure 1**); this drop is mainly observed in southern locations. Finally, no differences in aridity index are observed between the data provided by WorldClim 1 and WorldClim 2 for mountain papaya locations (**Figure 5**, insert).

The climatic behavior for growing locations of domesticated *F*. *chiloensis* f. *chiloensis* is shown in **Supplementary Figure 2**. Average annual temperature (BIO1) for the locations are the same in both models; however there is a reduction of about 1.7°C in the minimum temperature of the coldest month (BIO6) in WorldClim 2 compared to WorldClim1 (**Figure 6**). On the other hand, a dramatic reduction in rainfall is observed in southern locations (Purén) (around 100 mm) in WorldClim 2 compared to WorldClim 1 (**Supplementary Figure 2**). In general terms, there is a reduction in aridity index especially in the southern locations of domesticated strawberry (**Figure 6**, insert), which is also accompanied with a reduction in minimum temperatures during winter. As suggested by Bambach et al. (2013) these climate changes could affect the current distribution of the species.

Finally, the analysis of climate change for the locations of *F*. *chiloensis* f. *patagonica* is shown in **Supplementary Figure 3**. It is more complex to interpret as there is a latitudinal effect (North – South distribution of the localities) in addition to a longitudinal component (ie, East – West; mountain - seaside). Therefore, we will focus the comparison between the localities of Termas de Chillán and Cucao (**Figures 1** and **7**). Populations distributed between 36–40° South are located in the Andes mountain (nearby Termas de Chillán), meanwhile those distributed further south (42–46° South) are located at sea level (close to Cucao) (**Supplementary Figure 3**). A significant reduction in the minimum temperature of the coldest month of 4.2°C has been reported for Termas de Chillán location in WorldClim 2 compared to WorldClim 1 (**Figure 7**); a smaller reduction in minimum temperature was reported for Cucao (2.4°C) (**Supplementary Figure 3**). On the other hand, there are no significant differences in the average annual temperature for both locations, neither in the annual precipitation in Termas de Chillán (**Supplementary Figure 3**); nevertheless an increase of about 230 mm is reported in Cucao comparing WorldClim 2 and WorldClim 1. In terms of aridity index, there is a significant increment of around sixteen points for the location of Cucao in WorldClim 2 compared to WorldClim 1; a smaller increment in aridity index (4 points) was also observed in Termas de Chillán (**Figure 7**, insert). The particular climate properties of the Andes Mountains (high precipitation level, lower annual average temperature) could constitute a good refuge for this wild species, restricting current distributions to these areas (Bambach et al., 2013; Alarcón and Cavieres, 2015). In the case of cultivated species, the Andes Mountains can also constitute a refuge for the species, but its displacement to southern regions could be dedicated for agriculture development (Parajuli et al., 2019).

## Crop Potential

*Vasconcellea* species has an interesting potential as a new crop, considering potential breeding for fruit and other sub-products and its domestication in specific geographic areas (Scheldeman et al., 2011). The two most important species are the babaco (*Vasconcellea x heilbornii*) in Ecuador and Colombia, and mountain papaya (*Vasconcellea pubescens*), in all Andean countries and particularly important at small commercial scale in Colombia (Scheldeman et al., 2011) and Chile (Moya-León et al., 2004). Interestingly, the cultivated surface of mountain papaya almost disappears after the earthquake and Tsunami of 2010 in the Maule Region, mainly because natural growing areas of the species were salinized and damaged by seawater. In the Maule Region, toxic levels of salty soil inhibit the growth of *V. pubescens* plants, because the content of salt reduces water availability to plants. In addition, the reduction in soil pH in depth (1.4 units) and surface (0.7 units) resulted as consequence of the ionic strength generated and the coarse-textured soil (Casanova et al., 2016). Remarkably, an increase in the cultivated surface of this species has been recently observed in the northern extreme of Chile (Arica and Parinacota Region, ~ 18° 30' latitude South), where 13.6 ha were recorded that represents 9% of total cultivated surface for the species in Chile (ODEPA-CIREN, 2019).

*F. chiloensis* subsp. *chiloensis* has also potential as a new crop. Firstly, the production of phytochemicals with health benefits highlights the opportunity to use the species as a functional food. Secondly, the species has great resistance to several diseases (Rojas et al., 2013). Although Aphidborne virus (ABVs) has been detected in *F. chiloensis* subsp. *chiloensis* plants non visual symptoms of disease were determined, albeit the virus affects severely *F. vesca* plants. Similarly, *F. chiloensis* f *chiloensis* shows tolerance to *Botrytis cinerea*, a severe fungal disease which affects dramatically several fruit species (González et al., 2013). Also, it was reported that the Chilean strawberry presented higher tolerance to the fungus compared to *F*. × *ananassa*.

Ploidy and hybridization are the basis of current cultivated crops. Importantly, wild relatives have impacted positively the availability of crops, allowing the expansion of cultivation areas or increasing the tolerance to pest and diseases. In a climate change scenario, a breeding program is needed in order to obtain smart cultivars that can face the new challenges as a way to ensure sustainable production in the future (Borrell et al., 2020). Fruit homogeneity and other phenotypic characters have been the main effort of plant breeders, but new characteristic must be incorporated in modern crops, such as, tolerance to cold and high temperature environment, tolerance to drought or salinity and disease resistance (Doebley et al., 2006). May be taking into account the genetic variability of native species for those traits and using strategies like introgression or hybridization, the genetic improvement of these valuable crops can be possible.

Genomic selection has aided marker assisted selection to gain time during crop improvement (Heffner et al., 2010). Moreover, the incorporation of new tools for molecular breeding, such as genome sequencing, SNPs, or genome editing, facilitates the identification of new inbreeds; this strategy has been defined as design breeding (Heffner et al., 2011; Fernie and Yan, 2019).

Domestication of non-domesticated plants is a great opportunity to obtain cultivars well adapted to different edaphoclimatic niches. The long period of time required for selection from breeding programs can be accelerated by the use of a biotechnology approach and new genome editing techniques (Osterberg et al., 2017; Scheben et al., 2017; Wallace et al., 2018). Clearly, under the climate change scenario new type of cultivars are needed. The better adaptability to a variety of climatic conditions is a priority in current time as a way to reduce the negative impact of less food production in vulnerable environments.

Polyploidy can be a challenge for Chilean strawberry, but combining the information from genetic maps, the functional characterization of specific genes, and genome sequencing should be the starting point for applying this modern strategy (Rousseau-Gueutin et al., 2008; Davik et al., 2015; Vining et al., 2017; Edger et al., 2019; Moya-León et al., 2019). In the case of *Vasconcellea pubescens* there is no genomic or functional genomic data available until now, which can limit the breeding effort. However, wild plants are already adapted to a wide range of climatic conditions, considering their native habitat at high altitude of the Andes mountains. Chilean papaya is attractive as functional food and source of natural sub-products as papain proteases which can be the target for molecular breeding. For certain, the implementation of what is called breeding 4.0 or “*de novo* domestication” should provide new well adapted smart cultivars.

## Author Contributions

The present work was conceived and designed by RH, CG-E, LL, PP, and MM-L. LL and CG-E contributed collecting information and preparing figures. All authors contributed to the article and approved the submitted version.

## Conflict of Interest

The authors declare that the research was conducted in the absence of any commercial or financial relationships that could be construed as a potential conflict of interest.
