# Supplementary material for: Southern Species From the Biodiversity Hotspot of Central Chile: A Source of Color, Aroma, and Metabolites for Global Agriculture and Food Industry in a Scenario of Climate Change
Source: Front Plant Sci. 2020 Jul 2;11:1002. doi: 10.3389/fpls.2020.01002 (PMC7348657; doi:10.3389/fpls.2020.01002)
Supplement: Supplementary file 1 [file DataSheet_1.pdf]

Supplementary Table 1. List of the localities where southern crops were identified and used in climate analysis in WorldClim 1 and WorldClim 2.

N[a]	Specie	Locality	Lat.	Long.	Alt. (m asl)	Köppen-Geiger climate	Köppen-Geiger code
1	*V. pubescens*	La Serena	29° 58' 30.0" S	71° 13' 39.4" W	225	Cold Semi-arid climate with dry summer and oceanic influence	BSk (s) (i)
2	*V. pubescens*	Valle del Elqui	30° 00' 58.1" S	71° 01' 31.6" W	320	Cold Semi-arid climate with dry summer	BSk (s)
3	*V. pubescens*	Ovalle	30° 35' 43.8" S	71° 22' 30.3" W	270	Cold Semi-arid climate with dry summer	BSk (s)
4	*V. pubescens*	Huentelauquen	31° 34' 54.7" S	71° 31' 53.9" W	65	Cold Semi-arid climate with dry summer and oceanic influence	BSk (s) (i)
5	*V. pubescens*	La Ligua	32° 23' 35.5" S	71° 22' 28.9" W	35	Cold Semi-arid climate with dry summer	BSk (s)
6	*V. pubescens*	Lipimavida	34° 50' 48.9" S	72° 08' 30.8" W	20	Mediterranean climate (warm summer)	Csb
7	*V. pubescens*	Iloca	34° 55' 50.0" S	72° 10' 53.2" W	4	Mediterranean climate (warm summer)	Csb
8	*V. pubescens*	Curanipe	35° 50' 11.7" S	72° 37' 50.4" W	20	Mediterranean climate (warm summer) with oceanic influence	Csb (i)
9	*V. pubescens*	Cobquecura	36° 04' 18.8" S	72° 49' 9.90" W	20	Mediterranean climate (warm summer) with oceanic influence	Csb (i)
10	*V. pubescens*	Lleu-Lleu	38° 06' 47.9" S	73° 16' 53.9" W	50	Mediterranean climate (warm summer)	Csb
1	*F. chiloensis* f. *chiloensis*	Pelluhue	35° 50' 7.19" S	72° 35' 14.8" W	160	Mediterranean climate (warm summer) with oceanic influence	Csb (i)
2	*F. chiloensis* f. *chiloensis*	Huelon Alto	35° 05' 32.3" S	72° 03' 39.6" W	46	Mediterranean climate (warm summer)	Csb

3	*F. chiloensis* f. *chiloensis*	Llongocura	35° 10' 10.1" S	71° 59' 20.6" W	134	Mediterranean climate (warm summer)	Csb
4	*F. chiloensis* f. *chiloensis*	Purén	38° 03' 14.6" S	73° 11' 41.3" W	612	Mediterranean climate (warm summer)	Csb
1	*F. chiloensis* f. *patagonica*	Vilches Alto	35° 36' 29.9" S	71° 04' 3.20" W	1,220	Mediterranean climate (warm summer) with mountain influence	Csb (h)
2	*F. chiloensis* f. *patagonica*	Termas de Chillán	36° 55' 0.30" S	71° 27' 12.4" W	1,285	Mediterranean climate (warm summer) with mountain influence	Csb (h)
3	*F. chiloensis* f. *patagonica*	Cuesta Las Raices	38° 26' 22.4" S	71° 30' 38.9" W	1,200	Marine West Coast climate (warm and dry summer)	Cfb (s)
4	*F. chiloensis* f. *patagonica*	Termas Geométricas	39° 31' 50.8" S	71° 55' 51.8" W	692	Marine West Coast climate (warm and dry summer)	Cfb (s)
5	*F. chiloensis* f. *patagonica*	Curiñanco	39° 45' 17.2" S	73° 23' 23.3" W	5	Marine West Coast climate (warm and dry summer) with oceanic influence	Cfb (s) (i)
6	*F. chiloensis* f. *patagonica*	Cuesta Gutierrez	41° 19' 58.2" S	73° 17' 41.7" W	68	Marine West Coast climate (warm summer) with oceanic influence	Cfb (i)
7	*F. chiloensis* f. *patagonica*	Caulín	41° 48' 3.41" S	73° 40' 29.5" W	6	Marine West Coast climate (warm summer)	Cfb
8	*F. chiloensis* f. *patagonica*	Cucao	42° 38' 50.9" S	74° 06' 58.5" W	3	Marine West Coast climate (warm and dry summer)	Cfb (s)
9	*F. chiloensis* f. *patagonica*	Coyhaique	45° 32' 22.6" S	72° 00' 54.0" W	605	Mediterranean climate (mild summer)	Csc

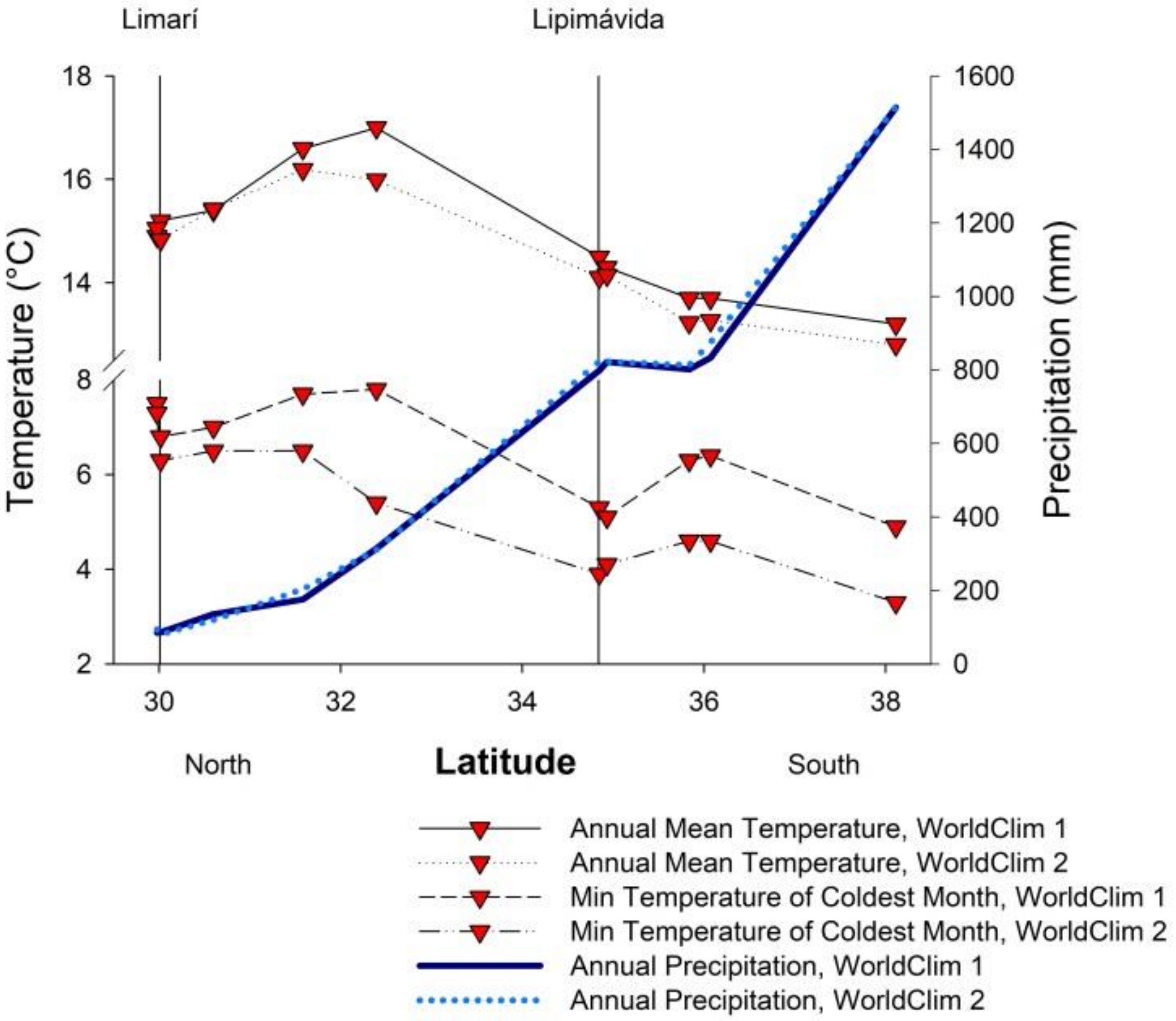

Supplementary Figure 1. Climatic characterization of cultivated localities for *V. pubescens* distributed from North to South. Bioclimatic variables of WorldClim 1 and 2 is shown. On the X axis, the localities of Limarí and Lipimávida are shown as reference, previously shown in Figure 1.

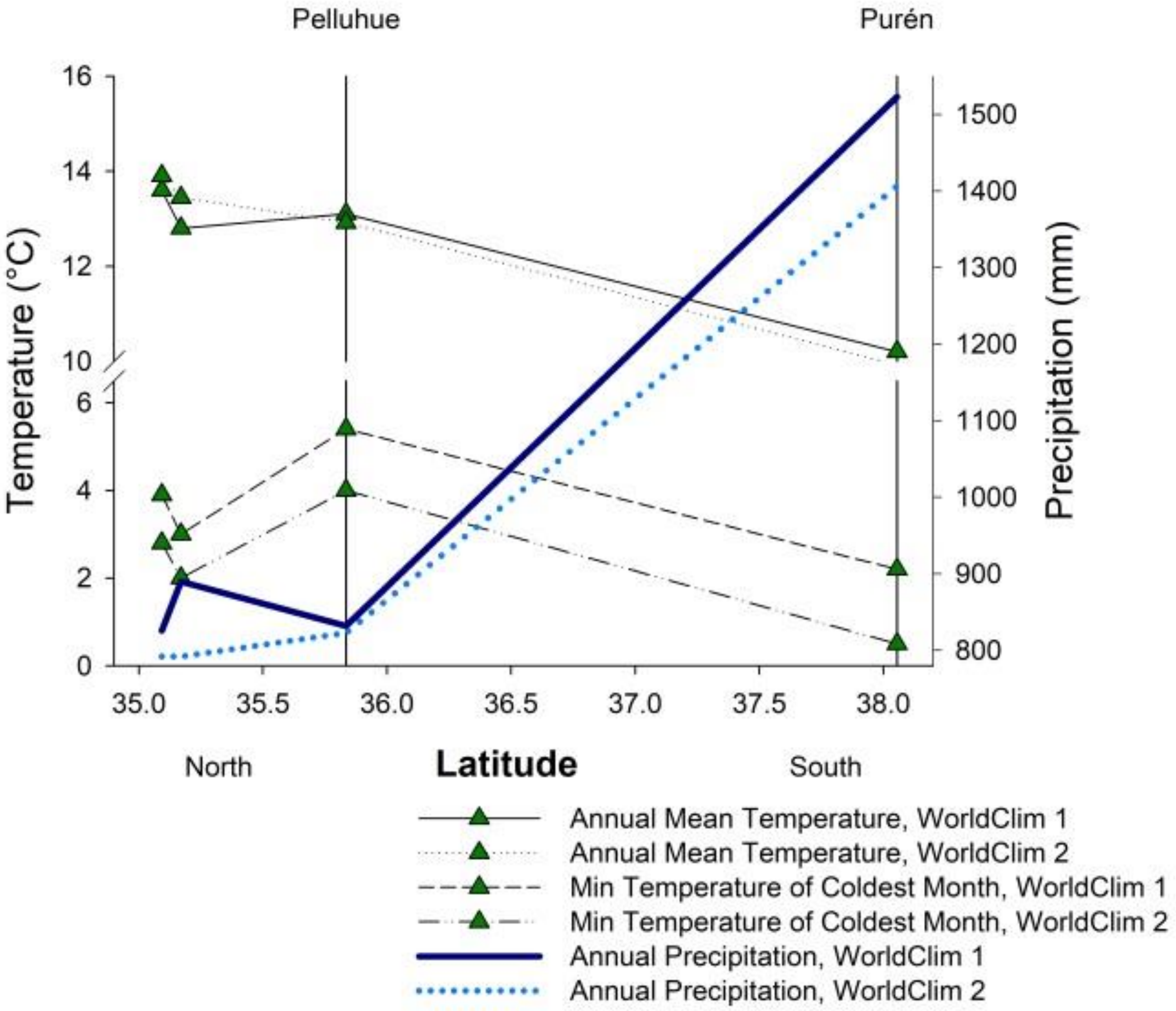

Supplementary Figure 2. Climatic characterization for the cultivated localities of *F. chiloensis* f. *chiloensis* distributed from North to South. Bioclimatic variables of WorldClim 1 and 2 is shown. The localities of Pelluhue and Purén are shown as reference in the X axis.

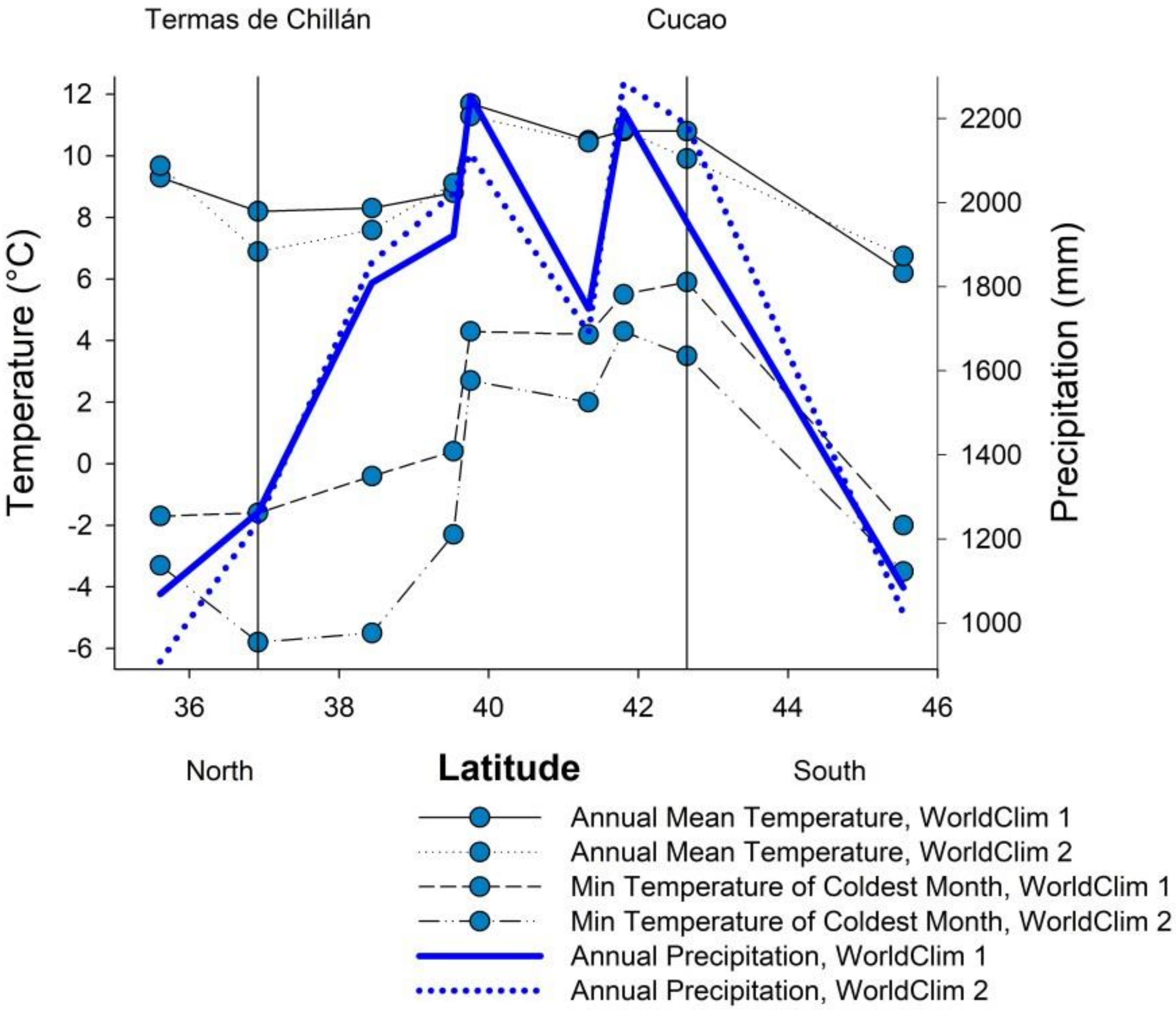

Supplementary Figure 3. Climatic characterization of the localities for wild *F. chiloensis* f. *patagonica* distributed from North to South. Bioclimatic variables of WorldClim 1 and 2 is shown. The localities of Termas de Chillán and Cucao are shown as reference in the X axis, which are previously shown in Figure 1.
